# Supplementary material for: The bacterial pH gradient contributes to persistence in Mycobacterium tuberculosis
Source: mBio. 2026 Aug 18;17(9):e01692-26. doi: 10.1128/mbio.01692-26 (PMC13556226; doi:10.1128/mbio.01692-26)
Supplement: Supplementary file 1 [file mbio.01692-26-s0001.docx]

**Supporting Information**

**The bacterial pH gradient and persistence in *Mycobacterium tuberculosis***

Hassan E. Eldesouky^1^, Kristin N. Adams^1^, Justin K. Brache^1^, Laarni Kendra T. Aguila^1^, Mariana Garcia^1^, Enming Xing^2,^ Pui-Kai Li^2^, and David R. Sherman^1*^

^1^ Department of Microbiology, University of Washington, Seattle, WA 98109, United States

^2^ Division of Medicinal Chemistry and Pharmacognosy, College of Pharmacy, The Ohio State University, Columbus, OH 43210, United States

*Correspondence: dsherman@uw.edu; Tel: +1(206)543-8152

**Supplementary Table 1**: Minimum inhibitory concentrations of different inhibitors against Mtb H37Rv strain.

| **MIC (μM)** | |
| --- | --- |
| Isoniazid (INH) | 0.25 |
| Rifampicin (RIF) | 0.005 |
| Ethambutol (EMB) | 0.25 |
| Pyrazinamide (PZA) | > 1000 |
| Amikacin (AMK) | 1 |
| Linezolid (LZD) | 3 |
| Moxifloxacin (MOX) | 0.15 |
| Pretomanid (Pa) | 0.2 |
| Bedaquiline (BDQ) | 0.5 |
| Telacebec (Q203) | 0.004 |
| Niclosamide (NCA) | 4 |
| Nic1 | 0.005 |
| Nic3 | 0.25 |
| Nic5 | > 1000 |
| Nic7 | 1 |
| Nic8 | 3 |
| Nic10 | 0.15 |
| Nic11 | 0.2 |
| Nic12 | 0.5 |
| Nic20 | 0.004 |
| Carbonyl cyanide 3 chlorophenylhydrazone (CCCP) | 8 |
| Nigericin (NIG) | 8 |
| Valinomycin (VAL) | 1 |

**Supplementary Figure 1: Effect of INH/RIF pretreatment on NCA-induced persistence.** *M. tuberculosis* H37Rv cultures were pretreated with INH/RIF (100× MIC) prior to exposure to niclosamide (NCA; 2× MIC) for 1, 3, or 24 h. Cultures treated with INH/RIF alone served as controls. Data represent the mean ± SD of three biological replicates. Statistical significance was determined by one-way ANOVA followed by Dunnett’s multiple-comparisons test (n = 3). Asterisks indicate significant differences relative to the control group.

**Supplementary Figure 2. Effect of niclosamide (NCA) on cell-associated rifampicin (RIF) concentrations in *M. tuberculosis* H37Rv.** Cells were treated with RIF (1 μg/ml) with or without NCA (2× MIC) for 1, 4, and 24 hours. Following treatment, cells were pelleted, washed twice with PBS, lysed by bead beating, and filter sterilized. RIF levels were quantified by mass spectrometry. Asterisks indicate statistical significance; *p*-values were determined using a two-tailed Welch’s *t*-test (n=3).

**Supplementary Figure 3*.*** **Effect of niclosamide (NCA) on rifampicin (RIF)-mediated intracellular killing of Mtb.** THP-1 cells were infected with M. tuberculosis H37Rv at a multiplicity of infection (MOI) of 1:1. Infected cells were then treated with RIF (1 μg/ml), NCA (0.25× MIC), or the combination of RIF and NCA. Data represent the mean percent survival of intracellular Mtb at day 4 post-infection ± standard deviation. Asterisks denote statistical significance; p-values were determined using a two-tailed Welch’s t-test (n=4).

**Supplementary Figure 4. Effect of NCA on antibiotic-mediated killing in other bacterial species. (a)** *E. coli* and **(b)** *S. aureus* were exposed to NCA (10 µg/ml or 0.25 µg/ml, respectively) or left untreated, alongside 100× MIC of amikacin (AMK), ceftazidime (CFT), or moxifloxacin (MOX).

**Supplementary Table 2:** Log2 fold changes of commonly regulated DEGs that were significantly altered (p < 0.02) by at least 2-fold in response to the tolerance-promoting inhibitors (NCA, CCCP, and NIG). Corresponding values from the VAL group are included for comparison, along with the average Log2 FC difference.

| **GENE_ID** | **Log2FC** | | | | **Average Log2 FC difference** **relative to VAL** |
| --- | --- | --- | --- | --- | --- |
|  | **NCA** | **CCCP** | **NIG** | **VAL** |  |
| Rv3290c | 3.91 | 2.58 | 3.28 | 0.72 | 2.53 |
| Rv3289c | 3.64 | 2.33 | 3.08 | 0.54 | 2.48 |
| Rv1057 | 2.80 | 1.05 | 1.52 | -0.87 | 2.66 |
| MT2165.1 | 2.43 | 1.28 | 2.97 | -0.91 | 3.14 |
| Rv2428 | 2.31 | 1.30 | 2.42 | 0.69 | 1.32 |
| Rv2107 | 2.27 | 1.16 | 3.29 | -0.08 | 2.32 |
| Rv2429 | 2.21 | 1.26 | 1.99 | 1.60 | 0.22 |
| Rv0211 | 1.74 | 1.21 | 1.26 | 0.83 | 0.57 |
| Rv1286 | 1.58 | 1.22 | 1.48 | 0.85 | 0.58 |
| Rv1285 | 1.38 | 1.04 | 1.57 | 1.58 | -0.25 |
| Rv0059 | -1.49 | -1.00 | -1.17 | 0.36 | -1.58 |
| Rv0241c | -1.77 | -1.08 | -1.73 | 0.81 | -2.34 |
| Rv0242c | -1.83 | -1.05 | -1.84 | 0.98 | -2.55 |
| Rv0057 | -1.95 | -1.15 | -1.17 | 2.91 | -4.33 |
| Rv2988c | -2.15 | -1.43 | -1.02 | 1.27 | -2.80 |
| Rv2987c | -2.46 | -1.10 | -1.01 | 2.15 | -3.68 |

**Supplementary Table 3.** PAM sequences, sgRNA sequences, and forward/reverse primer sequences for CRISPRi knockdown of selected genes.

| **Gene ID** | **pam Sequence** | **SgRNA target sequence** | **Forward primer** | **Reverse primer** |
| --- | --- | --- | --- | --- |
| Rv3290c | CGAGAAC | ATCAGCCCGCGGC  CGCGCGGAT | GGGAATCAGCCCGC  GGCCGCGCGGAT | AAACATCCGCGCGGC  CGCGGGCTGAT |
| Rv3289c | GCAGAAA | GCACGCCATACCC  GGTAGGTGC | GGGAGCACGCCATA  CCCGGTAGGTGC | AAACACGCGAAGTCG  AGGCCAC |
| Rv1057 | TGGGAAA | ATTGCGTGGTG  GTGCCCGGCGCC | GGGAATTGCGTGG  TGGTGCCCGGCGCC | AAACGGCGCCGGG  CACCACCACGCAAT |
| Rv2428 | CTGGAAA | GGTCGTTGTGCTG  TGCACGCCA | GGGAGGTCGTTGTG  CTGTGCACGCCA | AAACGATCACACACG  CCCCAAAGTT |
| Rv2107 | AAAGAAC | GGTACTGCTGCCC  GTGGCTACT | GGGAGGTACTGCTG  CCCGTGGCTACT | AAACCGAACTATTCG  GCCGGCGTTTGC |
| Rv0059 | GTAGAAC | GCATGGGCGACC  GCGCCGCAAT | GGGAGCATGGGCG  ACCGCGCCGCAAT | AAACATTGCGGCGCG  GTCGCCCATGC |
| Rv0241c | ATGGAAA | GCCGGTCACCAAC  GACATCACCG | GGGAGCCGGTCACC  AACGACATCACCG | AAACCGGTGATGTCGT  TGGTGACCGGC |
| Rv0242c | CAGGAAT | ACGTAGGCCGACT  TGGCCGACAG | GGGAACGTAGGCC  GACTTGGCCGACAG | AAACCTGTCGGCCA  AGTCGGCCTACGT |
| Rv0057 | CAAGAAT | AACGCGACGCCG  TTTGTGTGGA | GGGAAACGCGACGCCG  TTTGTGTGGA | AAACTCCACACAAA  CGGCGTCGCGTT |
| Rv2988c | CAAGAAC | GTGGGTGCGTGCG  GACGACCACG | GGGAGTGGGTGCGTG  CGGACGACCACG | AAACCGTGGTCGTCC  GCACGCACCCAC |
| Rv2987c | TCAGAAA | AAACCGGTTCGGGT  GACCCGCT | GGGAAAACCGGTTCG  GGTGACCCGCT | AAACAGCGGGTCACCCGA  ACCGGTTT |
